# Farmers’ Willingness to Participate in a Carbon Sequestration Program – A Discrete Choice Experiment

**DOI:** 10.1007/s00267-024-01963-9

**Published:** 2024-03-21

**Authors:** Julia B. Block, Michael Danne, Oliver Mußhoff

**Affiliations:** 1 Department of Agricultural Economics and Rural Development, University of Göttingen, Platz der Göttinger Sieben 5, 37073 Göttingen, Germany; 2 Thünen Institute, Institute of Farm Economics, Bundesallee 63, 38116 Braunschweig, Germany

**Keywords:** Carbon farming, Discrete choice experiment, German farmers, Humus programs

## Abstract

Farmers can counteract global warming by drawing carbon dioxide from the air into agricultural soils by building up humus. Humus programs were developed to motivate farmers for even more humus formation (= carbon sequestration) through an additional financial incentive. These programs are still at an early stage of development, which is why the number of participating farmers and research work is still low. This study is the first to analyze the willingness of German farmers to participate in hypothetical humus programs. The results of a discrete choice experiment show that a (higher) threshold for the payout of the premium, regional (rather than field-specific) reference values, and the risk of repayment clearly discourage farmers from participating. Program providers must more than double the premium (set at around 240 € per hectare and 0.1% humus increase) to maintain farmers’ willingness to participate despite a payout threshold. Regional reference values and an additional premium/repayment system would lead to an increase in the premium of around 20 € per hectare in order to keep the willingness to participate at the same level. The motivation to build up humus, the desire to maximize subsidies, and a higher livestock density have a positive influence on farmers’ decision to participate. Farm size and risk attitude have an impact on farmers’ preferences for program design. The study is relevant for policymakers and non-governmental organizations concerned with carbon management, as our findings highlight pathways for efficient, targeted designs of humus programs and carbon sequestration policies.

## Introduction

Agricultural soils have great potential to sequester carbon from the atmosphere (Janzen et al. 2022). They have the capacity to store 5% to 15% of global annual carbon emissions from fossil fuels (Lal, 2004). Reducing the concentration of carbon dioxide in the atmosphere is crucial to prevent excessive global warming and thus to achieve the global target of a maximum temperature increase of two degrees set in the Paris Agreement (Minasny et al. 2017; UNFCC 2015). Around half of the carbon stored in (German) ecosystems is stored in the top few meters of agricultural soil. Over the last 50 years, the carbon content in the soil has fallen considerably. In the years between 2012 and 2018, for example, the mineral soils used for arable farming suffered an average loss of organic carbon of 0.2 tons per hectare (ha) per year (Höper and Meesenburg 2021; Hüttl et al. 2008; Jacobs et al. 2018). Counteracting this development, i.e., preserving carbon in the soil and increasing carbon storage, is therefore becoming increasingly important in terms of climate protection.

Agricultural practices that aim to sequester carbon, also known as carbon farming, focus on building up humus in soils. Humus is formed by the decomposition of biomass, mainly plant residues. It is rich in carbon because plants absorb carbon dioxide from the air and convert it into organic matter (Chenu et al. 2019). Consequently, an increase in humus content corresponds directly to an increase in carbon sequestration. Carbon farming practices to increase humus content include e.g., intercropping, no-till, application of compost, and planting of new hedges or agroforestry systems (Sharma et al. 2021). Further advantages of higher humus content in the soil are higher soil fertility, improved nutrient storage and soil structure, increased water retention capacity, and thereby also erosion protection. Given the increasing frequency of extreme weather events due to climate change, these ecological benefits are likely to become even more important in the coming decades (Lal 2004). The ecological benefits of humus can also lead to economic benefits as it stabilizes yields under difficult weather conditions. Many farmers want to make an effort to increase the humus content of their fields, but there is a lack of clarity about which carbon farming practices are easy to implement and have a real impact on the climate. In addition, the costs of implementing the various measures vary greatly or are not known at all (Wüstemann et al. 2023).

To promote carbon sequestration in agricultural soils, non-governmental companies (and in some cases the government, e.g., in Australia and France) recently developed certification methodologies for carbon farming practices, so-called carbon sequestration programs. In the European Union (EU), there are around 20 different methodologies focused on agricultural land management that are widely used, most of them with an international reach (e.g., Agreena, Boomitra, Climate Farmers, Ormex, ReGeneration Soil Carbon, Sequana – Verra, Trinity NCM). Some of them relate only to soil carbon sequestration, while others include emissions from agriculture such as nitrous oxide emissions from soil or emissions from livestock. Many carbon sequestration programs specify which practices are eligible for carbon farming, such as reducing tillage (e.g., Climate Farmers), planting cover and catch crops (e.g., Agreena), direct seeding (e.g., ReGeneration Soil Carbon), and reducing the use of herbicides, insecticides (e.g., Trinity NCM) and nitrogen fertilizers (e.g., Ormex). There are roughly four approaches for quantifying soil carbon sequestration: using default factors from the literature, using a soil carbon model, using remote sensing, and using soil samples. Soil sampling is important to improve the modeling approaches and to verify the predicted changes in soil carbon (van Baren et al. 2023).

In contrast to carbon sequestration programs, participation in action-based Agri-Environmental Schemes (AES), adoption of organic farming, and specific carbon farming practices (e.g., mixed cropping, cover cropping) are well studied among farmers (Arbuckle and Roesch-McNally 2015; Bonke and Musshoff 2020; Buck and Palumbo-Compton 2022; Dessart et al. 2019; Dumbrell et al. 2016; Läpple and Kelley 2013; Paulus et al. 2022; Villamayor-Tomas et al. 2021). Kragt et al. (2016) investigated the adoption of carbon farming practices and participation in carbon farming policies among Australian farmers and uncovered barriers such as lack of information, uncertainty, and costs. Hannus et al. (2020) provide insights into the acceptance of sustainability standards by farmers and show that the required level of sustainability and the additional price premium are the most decisive characteristics. However, to our knowledge, there is no extant study that examines farmers’ preferences for the design of a carbon sequestration program (as described above), although such research would be needed to optimize programs and increase uptake and ultimately carbon stocks in soils. This is especially important given the early stage of development, as most carbon sequestration programs have only been on the market for a few years (e.g., Agreena since 2021, Boomitra since 2016, and Climate Farmers since 2020). The question of how to increase uptake is of particular interest to program providers and policymakers involved in carbon farming, as the EU, for example, is aiming for climate neutrality by 2050 (European Council 2023).

To gain first insights into the willingness of farmers to participate in a carbon sequestration program, we chose a specific certification methodology, which we henceforth refer to as ‘humus programs’. Humus programs, e.g., CarboCert (>440 German and Swiss farmers, founded in 2016), Ökoregion Kaindorf (402 Austrian and Slovenian farmers, founded in 2007), and Positerra (founded in Germany in 2019) operate on the private market and focus exclusively on increasing the humus content in agricultural soils, from which the increase in carbon sequestration can be derived. Their reach is relatively limited as they are often regionally restricted (e.g., Germany) and still under development.

Humus programs pay participating farmers a price premium if they increase the humus content within a certain period of time. This is financed through the sale of humus certificates, which are purchased by companies or private individuals. The premium is only paid if a considerable build-up of humus can be proven, a process which generally requires much more effort than farmers usually put in. In particular, the use of effective carbon farming practices that are rarely used due to their low ecological and economic benefit for agricultural production, such as the planting of new hedges, is to be promoted by the premium. The overarching goal of humus programs is thus to achieve considerably higher carbon storage levels than previously.

Humus programs use independently collected soil samples to quantify the humus accumulation, which is a valid strategy and reduces the complexity of our study. Another reason why we selected humus programs for our study is that these programs do not prescribe carbon farming practices and thereby meet farmers’ demand for fewer regulations but more incentive mechanisms. Farmers have full flexibility in increasing the humus content of their fields and can choose the carbon farming practices that suit them best in terms of topographical conditions, cultivation methods, and economic benefits. Humus program providers thus take into account studies that showed that farmers’ climate change adaptation intentions and willingness to participate in AES vary across regions due to farm and farmer characteristics (Espinosa‐Goded et al. 2010; Mitter et al. 2019). The adoption of certain sustainable management practices and the associated barriers are well studied, as noted above (see Dessart et al. 2019 for a review), but programs that focus on the outcome of carbon farming, regardless of which carbon farming practice has been adopted, are not. The aim of this study is therefore to determine the willingness of farmers to participate in humus programs. Specifically, we investigate for the first time whether, and to what extent specific program requirements as well as attitudinal and farm characteristics influence farmers’ willingness to participate in humus programs. In addition, we calculate the willingness to accept (WTA) to take into account the effects of influencing factors on the level of the price premium.

To achieve the objectives of the study, a discrete choice experiment (DCE) was conducted as an online survey with 150 German farmers in 2022. We deliberately chose German farmers as study participants because Germany occupies an important position in environmental protection, in the development of humus programs, and in scientific research on carbon farming. Germany is one of the most ambitious countries in terms of climate protection in the EU and plays a pioneering role in global climate strategies (Eurostat 2018; KAS 2016; Oberthür and Roche Kelly 2008; Parker and Karlsson 2010). In addition, the humus programs to which we refer are mainly developed and accessible in German-speaking countries. The topic is also of particular scientific relevance in Germany, where several research projects on carbon farming and humus formation are currently being carried out (HumusKlimaNetz 2022). The DCE was evaluated using a mixed logit model, as it considers the heterogeneity of preferences. With this approach, we build on previous studies in the field of agricultural and environmental research in which farmers’ preferences were estimated, e.g., for the design of AES (Bougherara et al. 2021; Ruto and Garrod 2009) or their willingness to participate in grazing programs (Danne and Musshoff 2017).

The remainder of this study is structured as follows: First, we derive factors that potentially influence farmers’ willingness to participate in a humus program. Then, the design of the DCE, the econometric modeling as well as the data collection are presented. Afterwards, we discuss the results, before we close with our conclusions and prospects for further research.

## Potential Factors Influencing Participation in Humus Programs Derived from Existing Programs and Literature on Sustainable Agriculture

Since program design can influence farmers’ willingness to participate, this section takes a closer look at existing humus programs and presents the individual program requirements chronologically. We point to the potential influence of program requirements on participation by referring to studies that examined farmers’ willingness to participate in carbon farming policies and AES, as well as the adoption of organic farming and certain carbon farming practices (Dessart et al. 2019; Hannus et al. 2020; Kragt et al. 2016; Läpple and Kelley 2015; Ogieriakhi and Woodward 2022; Paulus et al. 2022). AES are close to humus programs in terms of operating principles as farmers are compensated for income losses associated with the adoption of more appropriate farm management strategies to protect and enhance biodiversity (Batáry et al. 2015; Lécuyer et al. 2022). However, study results on the acceptance of AES are not fully transferable to humus programs, as humus programs are administrated on the private market and rely on results-based payments.

At the beginning of a humus program, soil samples are taken to determine the current humus content (in %) on the corresponding fields, which later serves as a reference value for detecting an increase in humus (Ökoregion Kaindorf 2022; Positerra 2022). With field-specific reference values, farmers who are already doing a lot to promote the humus content of their soil and thus can only implement a few (or no) further measures, will find it difficult to achieve a further substantial increase in the humus content and the resulting payment of a premium. At the same time farmers who have not yet made any effort to build up humus have great potential to increase the humus content and could receive high premiums. In addition, field-specific soil sampling is time-consuming, costly, and can contain measurement errors, which is why other quantification approaches such as remote sensing and soil carbon models came into play. However, soil sampling remains the most reliable method for measuring carbon sequestration and is often used in carbon sequestration programs, e.g., in combination with soil carbon models to improve and validate the models (van Baren et al. 2023). To reduce the number of field-specific soil samples, humus programs could use regional averages as reference values at the beginning by taking soil samples from regional reference farms. A regional reference value depends mainly on the soil composition and soil management of the farmers in the region and most likely does not correspond exactly to the individual humus content of each farmer. This could mean that farmers must either build up more humus than expected if they start with a low humus content but the regional value includes farms with a high humus content or that they must not build up much if they start with a high humus content but the regional value includes farms with a low humus content. The latter would allow for windfall profits sometimes attributed to carbon sequestration programs (Ogieriakhi and Woodward 2022). Since regional reference values are associated with more uncertainty for the individual farmer, we hypothesize the following:

H1: Farmers’ willingness to participate in humus programs increases ceteris paribus if the reference value is field-specific and not regionally defined.

At a certain time after humus programs recorded the reference value, a so-called success investigation takes place to prove whether the humus content has increased. More than half of the carbon sequestration programs have a duration of 10 years or less (van Baren et al. 2023). Farmers in humus programs usually have three to seven years until first control to increase the humus content through individually selectable carbon farming measures (Ökoregion Kaindorf 2022; Positerra 2022). During the success investigation, the humus content is measured and compared with the reference value. In case of an increase in the humus content, this is remunerated. Longer program durations are advocated by policymakers and society to ensure an unambiguous and stable humus build-up. Research on program duration is important to provide useful insights to help policymakers in their efforts to keep farmers longer in such programs (Defrancesco et al. 2018). Humus programs with longer periods give farmers more time to build up humus, which could lead to higher remuneration. At the same time, longer periods carry a higher risk of setbacks, e.g., due to weather conditions. In addition, the long planning horizon could discourage farmers from participating, so shorter periods could be more favorable. Nevertheless, we expect that farmers prefer longer periods, as humus build-up takes time, and hypothesize the following:

H2: Farmers’ willingness to participate in humus programs increases ceteris paribus with a longer period of time until the success investigation.

There are carbon sequestration programs that specify a minimum increase in humus growth, e.g., 0.2%, which must be achieved by the time of the success investigation in order to receive a payment. This is an attempt to circumvent the measurement error of 0.1% to 0.2% when measuring humus build-up and to ensure that farmers try to build up as much humus as possible (Kolbe and Zimmer 2015; Riedel 2020). The difference between humus content at the success investigation and reference value at the program start determines whether the farmers have achieved the minimum increase and receive a premium or not. Humus growth depends on agricultural practice, soil type, and existing humus content. We expect that farmers prefer a lower minimum increase as this is more promising to receive remuneration and hypothesize the following:

H3: Farmers’ willingness to participate in humus programs increases ceteris paribus with a lower minimum increase in humus content that must be achieved for payment.

Most existing humus programs pay a price premium of 30 € per ton of bound carbon dioxide for successful humus building by the time of the success investigation (Ökoregion Kaindorf 2022; Positerra 2022). Bartkowski and Bartke (2018) found that financial aspects are a key driver of land management change and participation in AES among farmers. The perception of the financial benefits that farmers associate with conservation tillage, organic farming, and low-emission practices also has a positive effect on acceptance (D’Emden et al. 2008; Läpple and Kelley 2015; Morgan et al. 2015). Therefore, we expect that an increasing price premium results in an increasing willingness to participate in humus programs and set up the following hypothesis:

H4: Farmers’ willingness to participate in humus programs increases ceteris paribus with a higher basic premium.

Humus programs carry out a second success investigation (=control investigation) to avoid the immediate degradation of humus components after payment of the price premium. The control investigation usually takes place three to five years after the success investigation. The programs use different approaches for the control investigation. Positerra (2022) pays out only two-thirds of the price premium after the success investigation and the remaining part after the control investigation if the humus content has at least not decreased. Ökoregion Kaindorf (2022) pays out the entire price premium at the success investigation but demands a proportional repayment if the humus content has decreased by the time of the control investigation. Another option could be the combination of an additional premium and a repayment at the time of the control investigation. Farmers receive an additional premium if the humus build-up has remained at least the same until the control investigation. Alternatively, the farmers must pay back the price premium proportionally if the humus content has decreased by the time of the control investigation. An additional premium attracts farmers to participate, but the simultaneous risk of having to pay something back (repayment) could instead discourage farmers from participating. Since an additional price premium can be an important driver for the adoption of sustainability standards (Hannus et al. 2020), AES (Wąs et al. 2021), and sustainable agricultural practices (Dessart et al. 2019) by farmers, we expect that the chance of an additional premium with the risk of repayment is preferable to no chance of additional compensation at all. We derive the following hypotheses:

H5: Farmers’ willingness to participate in humus programs increases ceteris paribus with the mechanism of an additional premium/repayment system.

In addition to program design, personal and farm characteristics can influence farmers’ participation. Studies show that farm size, land ownership, age, and education have a positive influence on participation in AES, while livestock density has a negative influence on participation (Breustedt et al. 2013; Paulus et al. 2022; Vanslembrouck et al. 2002; Wilson and Hart 2000). Prior knowledge of sustainable practices has a positive impact on the acceptance of carbon farming policies, organic farming practices, and participation in AES (Kragt et al. 2016; Läpple and van Rensburg 2011; Pavlis et al. 2016). Perceived risks have a negative influence on the introduction of conservation tillage and participation in AES (Kurkalova et al. 2006; Pavlis et al. 2016). Farmers who are concerned about the environment are more likely to adopt organic farming practices (e.g., Läpple and Kelley 2015). A positive attitude towards environmental issues is also one of the main reasons why farmers participate in the AES (Defrancesco et al. 2018; Lastra-Bravo et al. 2015; Vanslembrouck et al. 2002; Villamayor-Tomas et al. 2021; Wąs et al. 2021). Against this background, we expect that personal and farm characteristics influence farmers’ willingness to participate in humus programs and hypothesize the following:

H6: Socio-economic variables as well as farmers’ operational goals and environmental attitudes influence their willingness to participate in humus programs and their preferences for design.

## Research Methodology

### Discrete Choice Experiment Design

A DCE makes it possible to derive farmers’ preferences for humus program design from hypothetical decision-making situations. Since the acceptance of humus programs is still low, we could not derive the preferences from real decision-making situations, which is why a DCE is a suitable method in the context of our study (List et al. 2006; Louviere et al. 2000).

A DCE confronts the participants with several decision situations (=choice sets) consisting of different alternatives. Each choice set provides two exclusive alternatives and the opt-out option. We neutrally designated the two alternatives as ‘Humus Program A’ and ‘Humus Program B’ and added the opt-out option ‘no participation’ to each decision situation in order to avoid forced decisions and inconsistencies with the demand theory (Hanley et al. 2001).

Predefined attributes and associated levels describe each alternative. We selected the attributes and corresponding levels on the basis of existing humus programs (see previous section), expert advice, and a pilot study with 15 farmers who answered a preliminary DCE. The pilot study helped to reduce task complexity by sorting out unnecessary attributes and levels. This increases the feasibility of the experiment and reduces unobserved variability (DeShazo and Fermo 2002; Lancsar and Louviere 2008; Louviere et al. 2008). The influence of attributes and their levels on the selection decisions can be identified by varying them systematically across the choice sets (Louviere et al. 2000). Table 1 shows the attributes and levels of our final DCE, which we describe in more detail below.

**Table 1:** Attributes and Attribute Levels

Attributes	Levels
*Reference value* for the measurement of the humus increase	field-specific humus content at the start of the program | field-specific average of the last 3 years | regional average of the last 3 years
Timing of the *success investigation*	3 years | 5 years | 7 years
*Minimum increase* in humus content at success investigation	0.3% | 0.4% | 0.5%
*Basic premium* per 0.1% humus increase	180 €/ha | 200 €/ha | 220 €/ha | 240 €/ha
*Additional premium/repayment* per 0.1% humus increase/reduction at control investigation	50 €/ha | 0 €/ha

At the beginning of the humus program, the *reference value* can be measured field-specifically (as in most existing humus programs), or calculated using field-specific averages over time or regional averages over time and space. Field-specific reference values at the beginning of the program entail the risk of an outlier in the start year, e.g., due to specific weather events. This risk can be reduced if a field-specific average of the last three years is used as a reference value at the beginning. However, this requires own data from previous years, and windfall effects are possible, e.g., if farmers reduce their commitment to humus conservation/building in the years before the start of the humus program. Regional reference values have the advantage that no elaborate field-specific measurements are necessary at the start of the program or even in advance, but the individual reference is lost. How difficult it is for farmers to achieve a higher humus content than the regional average depends on various factors. The level of the regional average is determined, for example, by structural conditions such as soil type and soil quality within a region. It also depends on whether the farmers in the region are already doing a lot to improve the humus content or not. If a farmer has particularly humus-rich soils compared to the average for the region, windfall effects can arise: Without changing anything, the farmer receives a bonus for a higher humus content. The soil samples needed for the field-specific reference values are free of charge in our hypothetical humus programs. This enables a cost-independent comparison between all three levels.

The next step is the *success investigation*, by which time farmers must have achieved a *minimum increase* in humus formation in order to receive the price premium (=*basic premium*). The success investigation takes place 3 years, 5 years, or 7 years after the start of the program (comparable to existing humus programs). We set the minimum increase at 0.3%, 0.4%, or 0.5% as the potential of humus growth is estimated at 0.1% to 0.2% per year (CarboCert 2022). Farmers receive the basic premium once for the total humus increase per 0.1% humus growth if they have reached or even exceeded the minimum increase in the success investigation. The price premium of 30 € per ton of bound carbon dioxide in most existing humus programs corresponds to about 240 €/ha per 0.1% increase in humus. We derived four levels in steps of 20: 180 €/ha; 200 €/ha; 220 €/ha; 240 €/ha per 0.1% humus increase. We set 240 €/ha as the upper limit because the farmers in our hypothetical programs are financially relieved in terms of the costs for the soil samples (which they would have to pay in reality) and there is also the possibility of an additional premium (see next attribute).

Three years after the success investigation, a control investigation takes place, in which farmers either receive an *additional premium* or must *repay* the basic premium proportionally, depending on the humus content. The additional premium/repayment approach shows a new way to motivate farmers for steady humus growth. Farmers receive an additional premium (must make a repayment) if the humus content is above (below) the minimum increase that was already required at the time of the success investigation to receive the basic premium. The humus content in the control investigation is therefore compared with the minimum increase, and not with the humus content actually achieved in the success investigation, or the reference value from the start of the program. After the pilot study, we deliberately decide to offer only two levels for this attribute: either an additional premium/repayment of 50 €/ha or 0 €/ha. In this way, we do not overburden the participants and clearly distinguish this attribute from the price attribute (basic premium) in order to avoid problems in the evaluation. The level of 50 €/ha means that farmers receive an additional premium (must make a repayment) of 50 €/ha for every 0.1% humus content above (below) the minimum increase. If the humus content corresponds exactly to the minimum increase, neither an additional premium nor a repayment is due. The level of 0 €/ha means that the control investigation is carried out, but its result has no financial consequences.

We finally present the participants with 12 different choice sets consisting of two alternatives and five attributes (see Online Resource 4). The full-factorial design in fact leads to [$(3\bullet 3\bullet 3\bullet 4\bullet 2)$_Program A_ $\bullet \,(3\bullet 3\bullet 3\bullet 4\bullet 2)$_Program B_] = 46,656 possible choice sets. We reduced the number of choice sets to 12 by applying a D-efficient Bayesian design with prior parameters from the pilot study using the software ‘Ngene 1.1.2’. The number of 12 choice sets is consistent with Doherty et al. (2021), Kamphuis et al. (2015), and Mühlbacher and Bethge (2015). A D-efficient Bayesian design contains all possible choice sets and uses an algorithm that strives for a variance-covariance matrix with the smallest error term. Efficient designs take into account ex-ante information and associated uncertainties about random distributions of utility parameters by using prior parameter estimates from Bayesian parameter distributions (Rose and Bliemer 2009). We presented the choice sets to each participant in a random order. Table 2 shows an example of a choice set.

**Table 2:** Example of a Choice Set

Attributes	Humus Program A	Humus Program B	No Program Participation (opt-out)
Reference value for the measurement of the humus increase	Regional average of the last 3 years	Field-specific humus content at the start of the program	–
Timing of the success investigation in years	7	3	–
Minimum increase in humus content at success investigation	0.3%	0.4%	–
Basic premium per 0.1% humus increase	200 €/ha	220 €/ha	–
Additional premium/repayment per 0.1% humus increase/reduction at control investigation	50 €/ha	0 €/ha	–
Which alternative would you choose?	O	O	O

To ensure that every farmer understands our hypothetical humus programs, we used different types of explanations. We presented a detailed introductory text at the beginning, explaining the alternatives and attributes, as well as a timeline showing the process chronologically (see Online Resource 1). Four learning questions between the introduction and the experiment should help to understand the premium systems (see Online Resource 2). If participants answered a learning question incorrectly, they were given an information text leading to the correct answer before the next question was asked. Participants could access the explanations of the attributes and levels throughout the experiment by moving the cursor over the question mark buttons in the choice sets (see Online Resource 3).

### Econometric Modeling

Individuals choose the alternative for which they have the highest utility. According to random utility theory (Luce 1959; McFadden 1974), the utility $U$ of an individual *n* from choosing an alternative *s* contains a deterministic component *V*, and an independent and identically distributed (IID) random component ${\varepsilon }_{{sn}}$ (Hensher et al. 2015). The deterministic component can be divided into ${x}_{{sn}}$ as a vector of attributes and socioeconomic characteristics of *n*, and ${\beta }_{n}$ as a vector of individual parameters associated with ${x}_{{sn}}$:

1 $${U}_{{sn}}={V}_{{sn}}+{{\rm{\varepsilon }}}_{{sn}}={\beta }_{n}{x}_{{sn}}+{{\rm{\varepsilon }}}_{{sn}}$$

Under the assumption of utility maximization, the probability ${P}_{{sn}}$ that an individual *n* chooses alternative *s* instead of *j* from a finite set of choices ${C}_{n}$ is:

2 $${P}_{{sn}}={Prob}({U}_{{sn}} > {U}_{{jn}})\forall j\epsilon {C}_{n},s\,\ne\, j$$

In our analysis, we applied a mixed logit model^1^. The mixed logit model, also known as a random parameter model, is able to account for random variations in taste, which means that individuals have different *β*s. The utility parameters ${\beta }_{n}$ vary randomly across the sample population. Hence, the model considers preference heterogeneity, which strengthens the consistency with behavioral realism (Hensher et al. 2015; Train 2009). The choice probability in the mixed logit model is:

3 $${P}_{{sn}}={\int }_{\beta }\left(\frac{\exp \left({\beta }_{n}{x}_{{sn}}\right)}{{\sum }_{i}\exp \left({\beta }_{n}{x}_{{sn}}\right)}\right)f(\beta )d(\beta )$$

To consider the panel structure of the data set, we held random parameters constant over choice situations (Train 2009). Thus, Eq. (3) becomes:

4 $${P}_{{sn}}={\int }_{\beta }\left({\prod }_{t}\frac{\exp \left({\beta }_{n}{x}_{{snt}}\right)}{{\sum }_{s}\exp \left({\beta }_{n}{x}_{{snt}}\right)}\right)f(\beta )d(\beta )$$

where *t* = 1, … ,*T* contains the number of choice situations. The integral in Eq. (4) has no closed form and cannot be calculated exactly. Thus, the choice probability is approximated through the simulation of log-likelihood functions ${{LL}}_{n}$ determined by *R* simulation runs:

5 $${{LL}}_{n}={\sum }_{n}\mathrm{ln}\frac{1}{R}{\sum }_{r}{\prod }_{t}\frac{\exp \left({{\beta}^{\prime}}_{n}{x}_{{snt}}\right)}{{\sum}_{s}\exp \left({{\beta^{\prime}}}_{n}{x}_{{snt}}\right)}$$

To account for the heterogeneity of preferences in mixed logit models, we include individual-specific attributes in the model estimation process via interaction terms (Boxall and Adamowicz 2002; Hanley et al. 2003). We estimate the model using the software ‘Stata 14’ in conjunction with a *mixlogit* module with 1000 Halton draws (Hole 2007).

We further calculated the marginal WTA for the attributes. For this step, we divided the estimated attribute parameter of the variable in question by the estimated attribute parameter of the monetary variable (Hu et al. 2012; Schulz et al. 2014):

6 $${{WTA}}_{{X}_{k}}=-\frac{{\beta }_{k}}{{\beta }_{P}},$$

where ${\beta }_{k}$ and ${\beta }_{P}$ are the estimated coefficients of the attributes ${X}_{k}$ and the price *P*. We kept the parameters of the price attribute fixed (Das et al. 2009; Lancsar et al. 2017). We derive the WTA values and their confidence intervals by means of the Krinsky and Robb method^2^ using the Stata module *wtp* (Hole 2007) with 10,000 replications.

### Data Collection

For the empirical analysis, we collected primary data from German farm managers via an anonymous online survey in February and March 2022. We invited around 1000 farmers by email to take part in the survey voluntarily. The email addresses came from a mailing list that we collected in previous surveys, in which farmers explicitly expressed their interest in further surveys from us. In addition, we invited farmers via social media. The Ethics Committee and the Data Protection Supervisor of the University of Goettingen reviewed and approved the survey in advance. We structured the questionnaire as follows: First, farmers answered questions about the characteristics of their farm and carbon farming measures. Second, we implemented the introduction to the DCE, related learning questions, and the DCE itself (see Online Resource 1–4). Third, farmers answered questions on operating goals and climate change, which we took from the literature (e.g., Gramig et al. 2013; Greiner et al. 2009; Hyland et al. 2016), on a five-point Likert scale. Fourth, socio-demographic data was collected.

After removing incomplete questionnaires and checking for implausible responses, we were able to include 150 of the 272 answered questionnaires for the econometric analysis. According to ex-ante power calculations (Bartlett et al. 2001), a sample size of 150 corresponds to an allowable margin of error of 8% for the German farm population, assuming the usual confidence intervals. Many published DCEs with farmers provide small sample sizes, e.g., 49 U.S. farmers in Hudson and Lusk (2004), 97 English farmers in Beharry-Borg et al. (2013), 128 German farmers in Schulz et al. (2014), 104 French farmers in Jaeck and Lifran (2014), 104 Australian farmers in Greiner (2016), 165 German farmers in Fecke et al. (2018), and 90 French farmers in Chèze et al. (2020). A small sample size is a common limitation of DCE studies targeting farmers, as farmers are often hard to reach. The average processing time of the participants in our survey was 34 min^3^. In return, farmers who fully answered the questionnaire could choose between a gas station voucher and a construction market voucher worth 15 €.

## Results and Discussion

### Descriptive Results

The farmers in our sample partly correspond to the average German farmer in their descriptive statistics (see Table 3). Our sample is close to the German average farmer regarding conventional farming practices ([our sample:] 89% vs. [German average:] 90%), sex (94% male vs. 89% male), livestock (59 vs. 64%), and livestock density (1.03 vs 1.10 livestock units per ha). Clear differences exist in age (44 years vs. 53 years), education level (63% with university degree vs. 14%), arable land (180 ha vs. 64 ha), and share of rental land (47 vs. 60%) (German Farmers Association 2022; Statistisches Bundesamt 2021). Farmers in our sample are younger, more educated, and have larger farms than the German average. This is not representative of the present but could characterize a future sample against the background of structural change and more complex farm management. There are further differences in the regional distribution. Compared to the average distribution in Germany, twice as many farmers are represented in northern Germany in our survey. Eastern and western Germany, on the other hand, are appropriately represented. The average respondent has 21 years of farming experience and can be classified as risk-neutral on a scale from 0 (strongly risk averse) to 10 (strongly risk seeking) (Dohmen et al. 2011).

**Table 3:** Descriptive Statistics (*N* = 150)

Variable	Description	Mean/Share	S.D.	German Average^a^
*Age*	Farmers’ age in years	44.32	12.30	53.00
	≤25 years	0.01	–	0.10
	>25 and ≤35	0.30	–	0.15
	>35 and ≤45	0.24	–	0.15
	>45 and ≤55	0.21	–	0.22
	>55 years	0.23	–	0.37
*Education*	1, if the farmer holds a university degree; 0 otherwise	0.63	–	0.14
*Farming experience*	Farmers’ experience in years	20.95	14.18	n.a.
*Farm size*	Arable land in ha	180.95	279.56	64.10
	≤10 ha	0.08	–	0.25
	>10 and ≤20 ha	0.03	–	0.20
	>20 and ≤50 ha	0.13	–	0.23
	>50 and ≤100 ha	0.29	–	0.17
	>100 and ≤200 ha	0.26	–	0.10
	>200 and ≤500 ha	0.15	–	0.04
	>500 ha	0.07	–	0.02
*Farm type*	1, if the farm is managed conventionally; 0 otherwise	0.89	–	0.90
*Gender*	1, if the farmer is male; 0 otherwise	0.94	–	0.89
*Livestock*	1, if the farmer is engaged in livestock farming; 0 otherwise	0.59	–	0.64
*Livestock density*	Livestock density in livestock units per ha	1.03	0.95	1.10
*Maximum subsidies*	'Maximize premiums/subsidies’^b^	3.19	1.15	n.a.
*Motivation humus programs*	'Humus programs motivate me to accumulate humus in the soil.’^c^	3.22	1.14	n.a.
*Region*	Percent of farms in … states			
	North	43.30		20.30
	East	12.00		7.80
	West	20.00		24.80
	South	24.70		47.10
*Rental land*	Share of rental land in percent	46.86	27.87	60.00
*Risk attitude*	'Are you a person who is fully willing to take risks or do you try to avoid risks?’^d^	5.11	2.34	n.a.
*CO2-Certificates*	1, if the farmer knows humus certificates; 0 otherwise	0.89	–	n.a.
*Humus content*	1, if the farmer knows the humus content of their fields; 0 otherwise	0.37	–	n.a.
*Humus programs*	1, if the farmer knows humus programs; 0 otherwise	0.53	–	n.a.

^a^German average from the farmer population (German Farmers Association 2022; Statistisches Bundesamt 2021)

^b^On a Likert scale from 1 (very unimportant) to 5 (very important)

^c^On a Likert scale from 1 (reject completely) to 5 (agree completely)

^d^Risk attitude on a scale from 0 (strongly risk averse) to 10 (strongly risk seeking) according to Dohmen et al. (2011)

Maximizing subsidies is partly important for the farmers in our sample, and humus programs would partly motivate them to build up humus. They rated the farm objective ‘maximize premium/subsidies’ with an average of 3.19 on a Likert scale from 1 (very unimportant) to 5 (very important). The statement ‘humus programs motivate me to accumulate humus in the soil’ receives an average value of 3.22 on a Likert scale from 1 (reject completely) to 5 (agree completely) (see Table 3).

There is great potential to raise farmers’ awareness of humus building and humus programs and to attract farmers willing to participate. Only 37% of farmers surveyed know the humus content of their fields and can indicate their carbon sequestration potential. Only about half of the farmers in our sample (53%) have heard about humus programs. A lack of knowledge about the own humus content on the fields and about humus programs could be one of the main reasons why the number of farmers participating in humus programs is low. Deliberately increasing the humus content does not yet appear to be widespread. This could be related to the great uncertainty among farmers with regard to the implementation costs and climate impact of carbon farming measures (Wüstemann et al. 2023). Carbon dioxide certificates are known by nearly 90% of the surveyed farmers (see Table 3).

### Mixed Logit Model Results

#### Overview of the Hypotheses and their Results

Model 1 in Table 4 illustrates how the average farmer in our sample thinks about participating in humus programs and how the program attributes influence them. The variable ‘ASC’ (=Alternative Specific Constant) takes the value 1 if a farmer chose a humus program and the value 0 if they chose the option of non-participation. The coefficient of the ASC variable is positive (0.504), which indicates that farmers are generally willing to participate, but without statistical significance.

**Table 4:** Estimation Results of the Mixed Logit Model (*N* = 150)^a^

	Coefficients	
Variables	Model 1	Model 2
Program attributes		
ASC	0.504	−5.698***
Field-specific average of the last 3 years^b^	0.155**	0.146*
Regional average of the last 3 years^b^	−0.284**	−0.271**
Timing of the success investigation in years	0.115**	0.008
Minimum increase in humus content at success investigation	−5.047***	−7.163***
Basic premium per 0.1% humus increase	0.016***	0.016***
Additional premium/repayment of 50€/ha per 0.1% humus increase/reduction at control investigation^c^	−0.282***	−0.256***
Interaction terms		
ASC × Motivation humus programs		0.647**
ASC × Maximum subsidies		1.031***
ASC × Livestock density		0.758***
Timing of the success investigation × Farm size^d^		0.057***
Minimum increase × Farm size^d^		−0.728***
Minimum increase × Risk attitude^e^		0.515**
SD of random parameters		
SD ASC	4.359***	3.746***
SD field-specific average of the last 3 years^b^	0.490***	0.464***
SD regional average of the last 3 years^b^	1.037***	1.049***
SD timing of the success investigation in years	0.448***	0.447***
SD minimum increase in humus content at success investigation	3.068***	3.525***
SD additional premium/repayment of 50€/ha per 0.1% humus increase/reduction at control investigation^c^	0.639***	0.631***
Goodness of fit		
Participants/observations	150/1,800	150/1,800
Log-likelihood	−1308.44	−1292.35
AIC	2672.88	2652.70

Single, double, and triple asterisks (*, **, ***) indicate statistical significance at 10%, 5% and 1% level

^a^ASC = alternative specific constant; SD = standard deviation (The signs of estimated standard deviations are irrelevant and interpreted as being positive.); AIC = Akaike information criterion

^b^Effect-coded variable; base level is ‘field-specific humus content at start of the program’

^c^Effect-coded variable; base level is ‘0 €/ha’

^d^Arable land in 100 ha

^e^Risk attitude on a scale from 0 (strongly risk averse) to 10 (strongly risk seeking) according to Dohmen et al. (2011)

Farmers prefer field-specific reference values, longer program durations, and higher basic premiums. The statistically significant coefficients for the reference values show that farmers’ utility increases if they choose a humus program with a field-specific average value of the last three years (0.155). The utility decreases by choosing a humus program with a regional average value of the last three years (−0.284). Both values compare to a humus program with a field-specific humus content test taken at the beginning of the program. The results support H1, as we expected that a field-specific reference value would increase farmers’ willingness to participate compared to a regional reference value (see Table 5 for an overview of the hypotheses and our results). Furthermore, farmers’ utility increases statistically significantly by 0.115 with each additional year between the starting point and the success investigation. This is in line with our expectation formulated in H2 that farmers favor longer program periods. A statistically significant increase in farmers’ utility is also found with an increase in basic premium (0.016), which supports H4.

**Table 5:** Summary of Model Results

Hypotheses		Expected effect on participation	Mixed logit model 1	Mixed logit model 2
H1	Field-specific reference value	+	✓	✓
H2	Longer timing until success investigation	+	✓	n.s.
H3	Lower minimum increase	+	✓	✓
H4	Higher basic premium	+	✓	✓
H5	Additional premium/repayment	+	✓*	✓*
H6	Socio-economic variables/operational goals/environmental attitudes	explorative	—	✓

+ increase; - reduce

✓ statistically significant result

* different effect than expected

n.s. not statistically significant result

A higher minimum increase and the mechanism of an additional premium/repayment negatively influences farmers’ willingness to participate in humus programs. A 0.1% increase in the minimum increase reduces farmers’ utility statistically significantly by 5.047. The minimum increase has a particularly large influence on the participation decision, as the coefficient is about five times higher than the other coefficients. The result supports H3, according to which we expected that farmers would prefer a lower minimum increase. Farmers’ utility decreases statistically significantly by 0.282 if they opt for a humus program with an additional payment/repayment of 50 €/ha compared to a humus program without an additional premium/repayment. This result is not in line with our expectations formulated in H5, as we expected that farmers would prefer the chance of an additional premium with the risk of repayment to no chance of additional compensation at all.

Model 2 (see Table 4) shows interactions between additional covariates from the survey and the random coefficients from Model 1 to potentially explain the observed heterogeneity. Model 1 indicates heterogeneity around the mean of all program attributes, as the standard deviations (SDs) are statistically significant. All attributes, with the exception of the price variable, enter the model as random variables. We set the price variable ‘basic premium per 0.1% humus increase’ fixed for the calculation of the WTA and therefore no SD is estimated. To analyze possible interrelationships between the random variables, we estimate a model allowing for correlations.

We found a statistically significant influence of attitudinal and farm characteristics on farmers’ overall willingness to participate and on program attributes (see Model 2). This supports our expectation in H6 that farmers’ decisions on humus programs are also influenced by aspects other than program design. Farmers’ utility for participating in humus programs increases statistically significantly with increasing motivation for humus growth (0.647), increasing maximization of subsidies (1.031), and higher livestock density (0.758). Farm size is positively related to the duration of the humus establishment phase (0.057), but negatively related to the minimum increase (−0.728). Both coefficients are statistically significant. A risk-taking attitude has a positive and statistically significant influence on the minimum increase (0.515).

Numerous socio-demographic variables (e.g., age, education), farm characteristics (e.g., soil quality, precipitation, share of rented land), and other variables collected (e.g., knowledge of current humus content, perceived impacts of climate change) did not have a statistically significant coefficient and were therefore excluded by us. This is in line with DCE theory (Hensher et al. 2005), which states that statistically non-significant interaction terms should be excluded, as these could have an impact on all other parameter estimates of the model.

The coefficients and statistical significance level may differ between Model 1 and Model 2. Model 2 divides farmers’ utility for each attribute into different components through interaction terms. Hence, the coefficients differ from those in Model 1. The general utility behind the coefficients does not change. The statistical significance depends not only on its own influence on the outcome variable, but also on how it interacts with the other variables in the model when interaction terms are considered. The inclusion of interaction terms in Model 2 may therefore change the statistical significance level.

Based on the results in Table 4, we calculated the WTA for all statistically significant coefficients in Model 1 and Model 2 (see Table 6). The WTA results are discussed in more detail in the following sections.

**Table 6:** Farmers’ Willingness to Accept (*N* = 150)

	Model 1		Model 2	
Variables	WTA (€/ha)	Confidence intervals	WTA (€/ha)	Confidence intervals
Program attributes				
Field-specific average of the last 3 years^a^	9.93	[1.94; 18.75]	8.98	[1.01; 17.40]
Regional average of the last 3 years^a^	−18.21	[−31.41; −6.04]	−16.65	[−29.32; −4.59]
Timing of the success investigation in years	7.39	[2.30; 13.07]	0.50	[−5.02; 6.15]
Minimum increase in humus content at success investigation	−323.76	[−419.17; −247.27]	−439.70	[−622.54; −275.92]
Additional premium/repayment of 50€/ha per 0.1% humus increase/reduction at control investigation^b^	−18.10	[−26.58; −10.42]	−15.70	[−24.15; −8.34]
Interaction terms				
ASC × Motivation humus programs			39.72	[6.52; 74.65]
ASC × Maximum subsidies			63.29	[37.27; 94.39]
ASC × Livestock density			46.53	[19.73; 77.42]
Timing of the success investigation × Farm size^c^			3.50	[1.80; 5.42]
Minimum increase × Farm size^c^			−44.72	[−72.60; −18.86]
Minimum increase × Risk attitude^d^			31.62	[8.71; 56.23]

The WTA values were calculated for all statistically significant coefficients from Table 4, which led to the exclusion of the ASC variable. (The price variable was set fixed.)

^a^Effect-coded variable; base level is ‘field-specific humus content at the start of the program’

^b^Effect-coded variable; base level is ‘0 €/ha’

^c^Arable land in 100 ha

^d^Risk attitude on a scale from 0 (strongly risk averse) to 10 (strongly risk seeking) according to Dohmen et al. (2011)

#### WTA of the Program Attributes

Model 1 in Table 6 shows that field-specific reference values increase farmers’ WTA for humus programs. An increase in the WTA is associated with a reduction in the required basic premium. A field-specific average reference value of the last three years reduces the basic premium by 9.93 €/ha. A field-specific reference value measured at the beginning of the program reduces the basic premium by 8.28 €/ha. The latter is calculated by forming the negative sum of the values for the field-specific and regional average $(8.28 = (-1)\,\bullet \,(9.93-18.21))$. The highly similar values of the two field-specific values indicate that farmers are almost indifferent between these two. Hence, the commonly used field-specific value (collected at the start year) could be replaced by a field-specific average value (calculated from the last three years) as a reference value at the beginning of the program. If field-specific data are available, the field-specific average has the advantage of reducing the risk of having an outlier in the baseline year.

Regional average reference values of the last three years reduce farmers’ WTA for humus programs. In order to maintain the farmers’ acceptance, the basic premium must be increased by 18.21 €/ha. This underlines the general preference of farmers for field-specific reference values. A regional reference value at the beginning of a humus program could lead to windfall profits but also carry the risk that even more humus has to be built up if the individual humus content is below the regional reference value, which could deter farmers. This could well play a role for the farmers surveyed, as more than half of them do not even know the humus content of their fields (see Table 3), making it impossible for them to assess whether their humus content is above or below the regional average. Future studies could test whether our result also applies to all farmers who know the humus content of their fields. Furthermore, we set soil sampling as free in our hypothetical programs to investigate farmers’ preferences for the type of reference value regardless of cost. However, the cost of field-specific soil sampling in real programs could shift farmers’ preferences and should be considered in further studies.

A longer window of opportunity for humus formation in humus programs increases farmers’ WTA. Each additional year between the start of the program and the success investigation reduces the required basic premium by 7.39 €/ha. For example, a time window for humus build-up of 5 years reduces the basic premium by 36.95 €/ha ($=\,7.39\,\bullet \,5$). The more time available for humus build-up, the higher the probability of achieving the minimum increase required for a payout and the greater the willingness of the farmers surveyed to participate. Extending the humus growing phase can be crucial for policymakers and private certification companies to make humus programs more attractive. This is in line with their intention to ensure long-term storage of carbon in the soil and more accurate measurements, for which 10 years is too short (van Baren et al. 2023). However, farmers’ preferences might be different if there is no minimum increase that needs to be achieved for a payout.

A higher minimum increase results in a lower WTA for humus programs. If the minimum increase rises by 0.1%, the basic premium must be increased by 323.76 €/ha. The very high increase in the basic premium means that setting up a (high) minimum increase to receive the basic premium leads to a sharp decrease in farmers’ WTA. Premiums in humus programs must be doubled to tripled if a minimum increase is required. It is advisable to look for other mechanisms that ensure a considerable increase in carbon sequestration (e.g., staggered premium rates) to avoid setting a minimum increase, but still address the problem of inaccurate measurement in small ranges of humus increase (Riedel 2020).

The mechanism of an additional premium/repayment in a humus program leads to a decrease in farmers’ WTA. This mechanism increases the basic premium by 18.10 €/ha. The literature found that farmers with clear financial interests are more willing to participate in AES and introduce e.g., conservation tillage, organic farming practices, and low-emission practices (D’Emden et al. 2008; Läpple and Kelley 2015; Morgan et al. 2015; Wąs et al. 2021). In our hypothetical humus programs, an additional premium also means a threat of repayment, while no additional premium means no threat of repayment. The farmers surveyed preferred to choose a humus program without an additional premium, rather than take the risk of having to pay something back. Hence, farmers weigh the threat of repayment higher than the prospect of an additional premium. This result is particularly interesting for the development of incentives for long-term farmer participation in carbon sequestration programs.

#### WTA of the Interaction Terms

Model 2 in Table 6 shows that farmers with a motivation to build humus have a higher WTA for humus programs. Farmers who feel motivated to build up humus through humus programs accept a 39.72 €/ha lower basic premium. This result is in line with Vanslembrouck et al. (2002) and Villamayor-Tomas et al. (2021) who found that farmers with optimistic expectations are more interested in environmental protection measures. Increasing farmers’ motivation to build up humus, e.g., by communicating the ecological and economic benefits of humus, could therefore increase the acceptance of humus programs.

Farmers seeking to maximize subsidies have a higher WTA for humus programs. By striving for this farm target, the required basic premium can be reduced by 63.29 €/ha. Our result is in line with the literature, which states that fair premiums and clear economic interests are the main reasons for the adoption of conservation tillage, organic farming practices, and low-emission practices, as well as for participation in AES (D’Emden et al. 2008; Läpple and Kelley 2015; Lastra-Bravo et al. 2015; Morgan et al. 2015; Wąs et al. 2021). Wilson and Hart (2000) emphasized that environmental concerns and financial interests are not necessarily exclusive when participating in AES. Often, both motives are considered equally important by farmers. We support this statement by showing that both environmental motivation and maximizing subsidies have a positive influence on the participation decision in humus programs.

Livestock density has a positive impact on farmers’ WTA for humus programs. Farmers with a higher livestock density accept a 46.53 €/ha lower basic premium. This contradicts the findings of Breustedt et al. (2013) and Chèze et al. (2020), who found that higher livestock density reduces the likelihood of participation probability in AES and that livestock farmers are less willing to change their farming practices. However, higher livestock numbers per ha are usually accompanied by more manure and straw per ha and probably by more intercropping. All of this contributes to increasing the humus content and may indicate that livestock farmers already have a high humus content. This can lead to windfall effects if the reference value is determined regionally at the beginning of a humus program and includes farms with low humus content. Conversely, if the reference value is determined field-specific, an almost humus-saturated soil is disadvantageous, as a considerable increase in the humus content, which is required for the premium to be granted, is nearly impossible.

Farmers with larger farms have a higher WTA for longer periods to accumulate humus. With each additional year between the start year and the success investigation, the basic premium for large farms can be reduced by 3.50 € per 100 ha. For example, the shortest period of 3 years reduces the basic premium by 10.50 €/100 ha. Larger farms probably need more time to build up humus because they have higher management and logistical effort. They probably also participate with more ha than smaller farms. Our result is consistent with the literature, as farmers with larger farms are more willing to participate in AES longer (Defrancesco et al. 2018; Paulus et al. 2022; Wilson and Hart 2000).

More arable land reduces farmers’ WTA for a minimum increase. With a 0.1% increase in the minimum increase, the basic premium for larger farms increases by 44.72 € per 100 ha. In addition to the time aspect, resources for increasing humus content are probably also scarcer on larger farms. They are likely to need more organic material than farms with fewer registered ha. In particular, the WTA of farmers with higher land shares decreases with a shorter humus build-up time and higher minimum increase rates.

A risk-taking attitude has a positive impact on farmers’ WTA for a minimum increase. Farmers with a higher risk tolerance accept a 31.62 €/ha lower basic premium if the minimum increase rises by 0.1%. Wąs et al. (2021) found that Polish farmers are more likely to participate in AES if they are risk-averse. This leads to the conclusion that AES are considered part of risk management as they provide a guaranteed payment amount. The opposite is to be expected when participating in humus programs. These do not promise a guaranteed payment, as payment depends on achieving the minimum increase. There are no payments for farmers’ humus cultivation efforts if the minimum increase is not achieved. The minimum increase is therefore associated with high risk for farmers and farmers who are more risk averse are less inclined to participate in humus programs. Studies showed that perceived (financial) risks also have a negative impact on the adoption of conservation tillage and the development of sustainable husbandry (Kurkalova et al. 2006; Trujillo-Barrera et al. 2016).

## Conclusions

Agriculture can counteract climate change by sequestering and storing carbon dioxide from the atmosphere in the humus layer of agricultural soils. In order to make better use of the high carbon storage capacity of soils, humus programs have recently been developed to motivate farmers to build up humus and thus sequester carbon. As humus programs are hardly represented on the market and the number of participating farmers is low, there is a need to investigate farmers’ preferences regarding the design of humus programs. The aim of this study was therefore to determine the willingness of farmers to participate in humus programs. More specifically, we analyzed for the first time the influence of individual program requirements, individual attitudes, and farm characteristics on farmers’ WTA for humus programs. For this purpose, we conducted a DCE with 150 German farm managers in 2022 and evaluated it with a mixed logit model.

To get an initial sense of what might influence farmers’ participation in humus programs and how our DCE should be designed, we referred to existing carbon sequestration programs and the literature on organic farming, specific carbon farming practices, and AES. Key findings from the literature related to our study are that economic benefits, environmental awareness, length of commitment, risk attitude, prior knowledge, farm size, livestock, age, and education influence farmers’ adoption of sustainable agricultural practices/programs (e.g., Dessart et al. 2019; Hannus et al. 2020; Kragt et al. 2016; Läpple and Kelley 2015; Ogieriakhi and Woodward 2022; Paulus et al. 2022; Pavlis et al. 2016; van Baren et al. 2023; Vanslembrouck et al. 2002).

The central result of our empirical analysis is the farmers’ preferences regarding the design of humus programs. Higher basic premiums and longer periods for humus build-up between the starting point and the success investigation encourage farmers to participate in the programs. Furthermore, farmers prefer humus programs with a field-specific reference value needed to determine the humus increment instead of a regional reference value. A minimum increase in humus content, which must be achieved for a payout in the success investigation, has a strong negative impact on farmers’ willingness to participate in humus programs. The chance of an additional premium at the control investigation, which is at the same time associated with a repayment risk, also leads to a decline in farmers’ willingness to participate.

The decision to participate in humus programs is also influenced by individual attitudes and farm characteristics. The motivation to build humus through humus programs, the intention to maximize subsidies, and higher livestock density positively influence farmers’ participation decision. Farmers with larger farms in particular favor longer humus build-up phases and lower minimum increases. A risk-taking attitude has a positive influence on the acceptance of a higher minimum increase.

The results of the study enable advice on the promotion of humus formation to be given to policymakers and non-governmental organizations involved in carbon farming. We provide first insights into farmers’ heterogeneous decision-making behavior regarding a carbon sequestration program. From our results, the following indications can be derived for an increase in the participation rate in humus programs, provided that the basic premium is not considerably increased: (1) Instead of taking additional soil samples at the start of the program, field-specific average values can be used to calculate the reference value (if data is available), but not regional average values. (2) The time window for humus build-up between program start and success investigation should be extended. (3) A minimum increase that must be achieved for a payout should not be set or should at least be kept very low. (4) An additional premium in connection with a possible repayment should not be used as a control instrument. (5) Humus programs should be better publicized, with particular emphasis on the environmental and financial benefits.

A limitation of our study is the lack of representativeness of our sample. Future studies should therefore investigate whether the results also apply to a larger, representative, and multinational sample. We have shown that farmers prefer longer program durations, but the maximum length of our hypothetical programs is 10 years, which according to van Baren et al. (2023) is too short to ensure long-term carbon storage and accurate measurements. The question therefore arises as to whether additional years will have a negative impact on farmers’ willingness to participate at some point. Another open question is whether farmers still prefer field-specific reference values to regional reference values if the field-specific soil samples are not free of charge. Future studies should investigate the extent to which possible windfall effects, which can occur in particular when regional reference values are used at the beginning, influence farmers’ willingness to participate in a carbon sequestration program and their actual efforts to build up humus. Furthermore, various strategies to motivate farmers to maintain the humus content they have built up should be tested. Approaches without the possibility of repayment, e.g., withholding a certain proportion of the basic premium and only paying it if the humus content is still maintained after a few years, could prove successful. It would also be interesting to know how much land farmers would participate with, whether they would contribute land that is extensively farmed, and what impact carbon farming in some areas has on the intensity of land use in other areas. To this end, future studies should survey farmers who actually participate in an existing humus program. A survey among participating farmers would also be interesting in order to determine the advantages and disadvantages of the programs, positive and negative experiences, and suggestions for improvements from humus experts.

### Supplementary information

Online Resource 1

Online Resource 2

Online Resource 3

Online Resource 4

Online Resource 5

Online Resource 6

## Data Availability

The datasets generated during and/or analyzed during the current study are not publicly available due to the protection of individual privacy under the European General Data Protection Regulation but are available from the corresponding author on reasonable request.

## Footnotes

1 The mixed logit model is more in line with behavioral realism than other discrete choice models. It relaxes the rigid assumptions associated with IID error terms and allows an interpretation of the influence of the mean and variance of a given variable through the structure of its parameter space (Jones and Hensher 2004). The mixed logit model fits our data much better than the standard multinomial logit model (compared by Log-Likelihood and Akaike information criterion). The results of the multinomial logit model are presented in Online Resource 5 for completeness.

2 The Krinsky and Robb method is more suitable than the delta method because it does not assume symmetrical distribution of the WTP (Hole 2007). Online Resource 6 presents the confidence intervals of the delta method for completeness.

3 The average processing time is calculated based on 149 participants, as there is one participant with an exceptionally long participation time, which would increase the average processing time to 54 min. As we did not find any implausible data of this participant, we did not generally exclude this participant from the analysis.

## Code Availability

The software used for the analysis is Stata 14. Code is available on request.
