# Supplementary material for: Farmers’ Willingness to Participate in a Carbon Sequestration Program – A Discrete Choice Experiment
Source: Environ Manage. 2024 Mar 21;74(2):332–49. doi: 10.1007/s00267-024-01963-9 (PMC11227454; doi:10.1007/s00267-024-01963-9)
Supplement: Supplementary file 1 — Online Resource 1 [file 267_2024_1963_MOESM1_ESM.docx]

**Online Resource 1**

# *Environmental Management*

# Farmers’ willingness to participate in a carbon sequestration program – a discrete choice experiment

Julia B. Block*, Michael Danne, Oliver Mußhoff

* Georg-August-University Göttingen

Department of Agricultural Economics and Rural Development

Platz der Göttinger Sieben 5

37073 Göttingen, Germany

[juliabarbara.block@uni-goettingen.de](mailto:juliabarbara.block@uni-goettingen.de)

Introduction to the DCE (translated from German into English)

Dear farmer

We are interested in your willingness to participate in humus programs. You will be presented with 12 decision-making situations in which you have the option of participating in Humus Program A or Humus Program B. If you do not wish to participate under the mentioned conditions, you can also choose the option of "no program participation".

First of all, we would like to explain the general conditions that are relevant for participation in a humus program. Please note that the framework conditions may differ from the humus programs with which you might already be familiar.

Please imagine that you have the real possibility to participate in a humus program. You are free to choose the number of hectares with which you would like to participate in the program.

At the start of the program, the **reference value** (= baseline value) for the humus content of the corresponding fields is recorded (see timeline, step 1). Depending on the program, either your farm-specific average of the last three years, the regional average of the last three years or a field-specific humus content determined at the start of the program is applied as a reference value. The humus content of your fields is raised free of charge by an independent and state-certified laboratory.

After a certain period of time, a **success investigation** is carried out, which measures the increase in humus (see timeline, step 2). The success investigation determines whether you have achieved the **minimum increase** in humus defined in the program and are therefore eligible for payment of the **basic** **premium**. This is based on the difference between the humus content measured at the time of the success investigation and the reference value determined at the program start. For every 0.1% humus growth, you will be paid a basic premium per hectare. However, no basic premium will be paid to you if you have not achieved the minimum increase defined in the program at the time of the success investigation. How you achieve humus growth is up to you. No measures are prescribed.

Three years after the success investigation, a **control investigation** takes place to check whether the additionally built-up humus has demonstrably been retained, increased, or degraded (see timeline, step 3). If the humus content in the control investigation is above/below the minimum increase already required to receive the basic premium, an **additional premium/repayment** is made, depending on the program. If an additional premium/repayment is provided for, you will receive an additional premium for every 0.1% increase in humus above the minimum increase, or you will have to make a repayment for every 0.1% decrease in humus below the minimum increase. If the humus content has not changed from the success investigation to the control investigation, neither an additional premium nor a repayment is made. If no additional premium/repayment is foreseen in the program, the control investigation takes place, but its result has no cash consequences.

Timeline

➂ **Control investigation** (always 3 years after the success investigation) & possible payment of the *additional premium* or *repayment* of the basic premium

➀ Program start with detection of the **reference value** for the humus content of the areas

Years

0 1 2 3 4 5 6 7 8 9 10

➁ **Success investigation** & payment of the *basic premium* (timing varies between 3 and 7 years after program start)
