# Supplementary material for: Farmers’ Willingness to Participate in a Carbon Sequestration Program – A Discrete Choice Experiment
Source: Environ Manage. 2024 Mar 21;74(2):332–49. doi: 10.1007/s00267-024-01963-9 (PMC11227454; doi:10.1007/s00267-024-01963-9)
Supplement: Supplementary file 2 — Online Resource 2 [file 267_2024_1963_MOESM2_ESM.docx]

**Online Resource 2**

# *Environmental Management*

# Farmers’ willingness to participate in a carbon sequestration program – a discrete choice experiment

Julia B. Block*, Michael Danne, Oliver Mußhoff

* Georg-August-University Göttingen

Department of Agricultural Economics and Rural Development

Platz der Göttinger Sieben 5

37073 Göttingen, Germany

[juliabarbara.block@uni-goettingen.de](mailto:juliabarbara.block@uni-goettingen.de)

Learning Questions (translated from German into English)

*Before the decision-making situations start, you will see a calculation example below that is intended to make the premium systems more understandable. Please answer the four corresponding questions. If you answer incorrectly, you will be shown an explanation and a guide to the correct answer. The numbers in the calculation example do not match the numbers in the later experiment.*

Please imagine that the reference value for the humus content of the corresponding fields recorded at the start of the program is 2 %. The minimum increase is given as 0.6 %. The basic premium after the success investigation is 100 €/ha per 0.1 % humus enrichment. The additional premium or repayment per 0.1 % humus enrichment or humus reduction after the control investigation is 50 €/ha.

Summary:

**Reference value** at program start: 2%

**Minimum increase** until success investigation: 0.6%

**Basic premium** at success investigation: 100 €/ha per 0.1% humus increase

**Additional premium/repayment** at control investigation: +/-50 €/ha per 0.1% humus increase/humus reduction

1. How much €/ha do you receive if the humus content of the corresponding fields is 2.8% at the time of the success investigation?

⃝ 200 €/ha

⃝ 400 €/ha

⃝ 800 €/ha

*🡪 Input if the answer is incorrect:* As the minimum increase is 0.6%, your fields must contain at least 2.6% humus at the time of the success investigation in order to receive a basic premium. Since the humus content at the time of the success investigation is 2.8%, you are above the minimum increase. This makes you eligible for payment of the basic premium. The basic premium is paid for every single 0.1% increase in humus content. In this case, you will receive 100 €/ha per 0.1% for the entire 0.8% humus build-up. This means that you receive a basic premium of 800 €/ha.

1. How much €/ha do you receive if the humus content is 2.3% at the time of the success investigation?

⃝ 0 €/ha

⃝ 300 €/ha

⃝ 600 €/ha

*🡪 Input if the answer is incorrect*: Since the minimum increase is 0.6%, your fields must contain at least 2.6% humus at the time of the success investigation. However, your fields only contain a humus content of 2.3% at the time of the success investigation. This means that you are not eligible for payment of the basic premium and receive 0 €/ha.

1. Assuming you have achieved a humus content of 2.8% in the performance test and thus you have exceeded the minimum increase (0.6%) by 0.2%. Accordingly, a basic premium was paid to you. Three years later, the control investigation is conducted. Now, your fields contain a humus content of 2.7%. Do you receive an additional premium or do you have to make a repayment?

⃝ Additional premium of 350 €/ha

⃝ Additional premium of 50 €/ha

⃝ Repayment of 350 €/ha

⃝ Repayment of 50 €/ha

*🡪 Input if the answer is incorrect:* In the control survey, the starting point is no longer the reference value determined at the start of the program, but the minimum increase already required to receive the basic premium. In the present case, you must have reached 2.6% humus content at the time of the success investigation. Regardless of whether you are above this at the time of the success investigation, the minimum value of 2.6% is the reference value for the control investigation. If the humus content at the time of the control investigation is above the minimum value, in this case above 2.6%, you will receive an additional premium of 50 €/ha for each 0.1% humus content above the 2.6%. If the humus content at the time of the control investigation is below the minimum value, in this case below 2.6%, you must pay a repayment of 50 €/ha for each 0.1% humus content below the 2.6%. If the humus content at the time of the control investigation was 2.6%, i.e. exactly the minimum value, you would neither receive an additional premium nor would you have to make a repayment. In this example, your humus content (2.7%) is 0.1% above the minimum value (2.6 %). Therefore, you will receive an additional premium of 50 €/ha.

1. Assume that the humus content of your fields at the time of the control investigation is not 2.7% but 2.5%. Do you receive an additional premium or do you have to make a repayment?

⃝ Additional premium of 250 €/ha

⃝ Additional premium of 50 €/ha

⃝ Repayment of 250 €/ha

⃝ Repayment of 50 €/ha

*🡪 Input if the answer is incorrect:* In the control investigation, the starting point is no longer the reference value determined at the start of the program, but the minimum increase already required to receive the basic premium. In the present case, you must have reached 2.6% humus content at the time of the success investigation. Regardless of whether you are above this at the time of the success investigation, the minimum value of 2.6% is the reference value for the control investigation. If the humus content at the time of the control investigation is above the minimum value, in this case above 2.6%, you will receive an additional premium of 50 €/ha for every 0.1% humus content above the 2.6%. If the humus content at the time of the control investigation is below the minimum value, in this case below 2.6%, you must pay a repayment of 50 €/ha for every 0.1% humus content below the 2.6%. If the humus content at the time of the control investigation was 2.6%, i.e. exactly the minimum value, you would neither receive an additional premium nor would you have to make a repayment. In this example, your humus content (2.5 %) is 0.1 % below the minimum value (2.6 %). Therefore, you have to make a repayment of 50 €/ha.
