# Supplementary material for: Farmers’ Willingness to Participate in a Carbon Sequestration Program – A Discrete Choice Experiment
Source: Environ Manage. 2024 Mar 21;74(2):332–49. doi: 10.1007/s00267-024-01963-9 (PMC11227454; doi:10.1007/s00267-024-01963-9)
Supplement: Supplementary file 3 — Online Resource 3 [file 267_2024_1963_MOESM3_ESM.docx]

**Online Resource 3**

# *Environmental Management*

# Farmers’ willingness to participate in a carbon sequestration program – a discrete choice experiment

Julia B. Block*, Michael Danne, Oliver Mußhoff

* Georg-August-University Göttingen

Department of Agricultural Economics and Rural Development

Platz der Göttinger Sieben 5

37073 Göttingen, Germany

[juliabarbara.block@uni-goettingen.de](mailto:juliabarbara.block@uni-goettingen.de)

Information buttons (translated from German into English)

*Additional information: Regional average of the last 3 years*

The regional average of the last 3 years can have advantages and disadvantages as a reference value at the start of the program. If the regional average (= 2 %) is, for example, 0.3 % below the humus content of your own land (= 2.3 %), you would actually only have to build up 0.3 % humus with a minimum increase of, for example, 0.6 % in order to achieve the minimum value (= 2.6 %) and thus the payment of the basic premium. However, if the regional average (= 2 %) is, for example, 0.3 % higher than the humus content of your own land (= 1.7 %), you would even have to build up 0.9 % humus by the time of the success test if the minimum increase is, for example, 0.6 %. If the regional average matches the humus content of your own land, there are neither advantages nor disadvantages.

*Additional information: Example of the humus premium*

Suppose you register your farm with 20 ha for the humus program and the minimum increase up to the success investigation is 0.6% humus build-up. At the time of the success investigation, a humus build-up of 0.8% can be verified on your land. This means that you have bound around 65 tons of CO_2_ per ha. With a humus premium of 30 €/t of bound CO_2_, the equivalent of around 240 €/ha per 0.1 % humus build-up, you will receive a basic premium of 1,920 €/ha. For 20 ha, this corresponds to a total basic premium of 38,400 €.

If you have built up a further 0.2 % humus at the time of the control investigation (a total of 1 % humus build-up from the start of the program), you will receive a total additional premium of 2,400 € with an additional premium of 60 €/ha per 0.1 % humus build-up above the minimum increase.

*Additional information: Field-specific humus content at the start of the program*

The field-specific humus content at the start of the program corresponds to the current humus content of the areas you have registered for the humus program. The humus content is determined free of charge via a soil sample by an independent and state-certified laboratory.

*Additional information: Field-specific average of the last 3 years*

The field-specific average of the last 3 years is determined from your own documentation on the humus content from the last 3 years. If the average is below the current humus content of your land, you must enrich less humus than officially stated in order to achieve the minimum increase. However, if the average is above the current humus content of your land at the start of the program, you must build up more humus than officially recorded in order to achieve the minimum increase.

*Additional information: Time of the success investigation*

During the success investigation, soil samples are taken from the relevant fields to check whether the specified minimum increase has been achieved. The time of the success test is between 3 and 7 years after the start of the program.

*Additional information: Minimum increase*

If the minimum increase is reached at the time of the success investigation, a basic premium is paid for each 0.1% increase in humus. If it is not reached, no basic premium is paid. The minimum increase serves as a reference value for the control investigation. If the humus content is above the minimum increase at the time of the control investigation, an additional premium is paid. If it is below, a pro rata repayment of the basic premium must be made.

*Additional information: Basic premium*

If the minimum increase is reached at the time of the performance test, a basic premium is paid for the entire increase in humus per 0.1 % of humus built up.

*Additional information: Additional premium/repayment*

If the humus content in the control investigation is above the minimum increase required to receive the basic premium, an additional premium is paid for each 0.1% increase in humus above the minimum increase. If the humus content is below the minimum increase, no additional premium is paid. Instead, a pro rata repayment of the basic premium must be made for every 0.1% below the minimum increase.

0 €/ha: The inspection takes place, but its result has no cash consequences.

50 €/ha: If you have built up further humus after the success investigation, you will receive an additional premium of 50 €/ha for each 0.1% increase in humus above the minimum increase after the control investigation. However, if you have depleted humus after the success investigation, you must make a repayment of €50/ha per 0.1% humus depletion below the minimum increase after the control investigation. If your humus content has remained the same from the success investigation to the control investigation, you will neither receive an additional premium nor will you have to make a repayment.
