# Supplementary material for: Farmers’ Willingness to Participate in a Carbon Sequestration Program – A Discrete Choice Experiment
Source: Environ Manage. 2024 Mar 21;74(2):332–49. doi: 10.1007/s00267-024-01963-9 (PMC11227454; doi:10.1007/s00267-024-01963-9)
Supplement: Supplementary file 4 — Online Resource 4 [file 267_2024_1963_MOESM4_ESM.docx]

**Online Resource 4**

# *Environmental Management*

# Farmers’ willingness to participate in a carbon sequestration program – a discrete choice experiment

Julia B. Block*, Michael Danne, Oliver Mußhoff

* Georg-August-University Göttingen

Department of Agricultural Economics and Rural Development

Platz der Göttinger Sieben 5

37073 Göttingen, Germany

[juliabarbara.block@uni-goettingen.de](mailto:juliabarbara.block@uni-goettingen.de)

Choice Sets (translated from German into English)

**Table 1:** Choice Set 1

| Attributes | Humus Program A | Humus Program B | No Program Participation (opt-out) |
| --- | --- | --- | --- |
| Reference value for the measurement of the humus increase | Regional average of the last 3 years | Field-specific average of the last 3 years | - |
| Timing of the success investigation in years | 5 | 5 | - |
| Minimum increase in humus content at success investigation | 0.4% | 0.3% | - |
| Basic premium per 0.1% humus increase | 180 €/ha | 240 €/ha | - |
| Additional premium/repayment per 0.1% humus increase/reduction at control investigation | 50 €/ha | 0 €/ha | - |
| Which alternative would you choose? | O | O | O |

**Table 2:** Choice Set 2

| Attributes | Humus Program A | Humus Program B | No Program Participation (opt-out) |
| --- | --- | --- | --- |
| Reference value for the measurement of the humus increase | Regional average of the last 3 years | Field-specific humus content at the start of the program | - |
| Timing of the success investigation in years | 7 | 3 | - |
| Minimum increase in humus content at success investigation | 0.3% | 0.4% | - |
| Basic premium per 0.1% humus increase | 200 €/ha | 220 €/ha | - |
| Additional premium/repayment per 0.1% humus increase/reduction at control investigation | 50 €/ha | 0 €/ha | - |
| Which alternative would you choose? | O | O | O |

**Table 3:** Choice Set 3

| Attributes | Humus Program A | Humus Program B | No Program Participation (opt-out) |
| --- | --- | --- | --- |
| Reference value for the measurement of the humus increase | Field-specific humus content at the start of the program | Field-specific average of the last 3 years | - |
| Timing of the success investigation in years | 3 | 7 | - |
| Minimum increase in humus content at success investigation | 0.5% | 0.5% | - |
| Basic premium per 0.1% humus increase | 200 €/ha | 200 €/ha | - |
| Additional premium/repayment per 0.1% humus increase/reduction at control investigation | 0 €/ha | 50 €/ha | - |
| Which alternative would you choose? | O | O | O |

**Table 4:** Choice Set 4

| Attributes | Humus Program A | Humus Program B | No Program Participation (opt-out) |
| --- | --- | --- | --- |
| Reference value for the measurement of the humus increase | Field-specific average of the last 3 years | Regional average of the last 3 years | - |
| Timing of the success investigation in years | 7 | 3 | - |
| Minimum increase in humus content at success investigation | 0.4% | 0.5% | - |
| Basic premium per 0.1% humus increase | 220 €/ha | 220 €/ha | - |
| Additional premium/repayment per 0.1% humus increase/reduction at control investigation | 0 €/ha | 50 €/ha | - |
| Which alternative would you choose? | O | O | O |

**Table 5:** Choice Set 5

| Attributes | Humus Program A | Humus Program B | No Program Participation (opt-out) |
| --- | --- | --- | --- |
| Reference value for the measurement of the humus increase | Field-specific average of the last 3 years | Regional average of the last 3 years | - |
| Timing of the success investigation in years | 5 | 5 | - |
| Minimum increase in humus content at success investigation | 0.4% | 0.5% | - |
| Basic premium per 0.1% humus increase | 220 €/ha | 200 €/ha | - |
| Additional premium/repayment per 0.1% humus increase/reduction at control investigation | 50 €/ha | 0 €/ha | - |
| Which alternative would you choose? | O | O | O |

**Table 6:** Choice Set 6

| Attributes | Humus Program A | Humus Program B | No Program Participation (opt-out) |
| --- | --- | --- | --- |
| Reference value for the measurement of the humus increase | Field-specific humus content at the start of the program | Regional average of the last 3 years | - |
| Timing of the success investigation in years | 5 | 5 | - |
| Minimum increase in humus content at success investigation | 0.5% | 0.3% | - |
| Basic premium per 0.1% humus increase | 240 €/ha | 180 €/ha | - |
| Additional premium/repayment per 0.1% humus increase/reduction at control investigation | 50 €/ha | 0 €/ha | - |
| Which alternative would you choose? | O | O | O |

**Table 7:** Choice Set 7

| Attributes | Humus Program A | Humus Program B | No Program Participation (opt-out) |
| --- | --- | --- | --- |
| Reference value for the measurement of the humus increase | Field-specific average of the last 3 years | Field-specific humus content at the start of the program | - |
| Timing of the success investigation in years | 3 | 7 | - |
| Minimum increase in humus content at success investigation | 0.4% | 0.3% | - |
| Basic premium per 0.1% humus increase | 180 €/ha | 240 €/ha | - |
| Additional premium/repayment per 0.1% humus increase/reduction at control investigation | 0 €/ha | 50 €/ha | - |
| Which alternative would you choose? | O | O | O |

**Table 8:** Choice Set 8

| Attributes | Humus Program A | Humus Program B | No Program Participation (opt-out) |
| --- | --- | --- | --- |
| Reference value for the measurement of the humus increase | Regional average of the last 3 years | Field-specific humus content at the start of the program | - |
| Timing of the success investigation in years | 3 | 7 | - |
| Minimum increase in humus content at success investigation | 0.3% | 0.4% | - |
| Basic premium per 0.1% humus increase | 240 €/ha | 180 €/ha | - |
| Additional premium/repayment per 0.1% humus increase/reduction at control investigation | 0 €/ha | 50 €/ha | - |
| Which alternative would you choose? | O | O | O |

**Table 9:** Choice Set 9

| Attributes | Humus Program A | Humus Program B | No Program Participation (opt-out) |
| --- | --- | --- | --- |
| Reference value for the measurement of the humus increase | Field-specific humus content at the start of the program | Field-specific average of the last 3 years | - |
| Timing of the success investigation in years | 3 | 7 | - |
| Minimum increase in humus content at success investigation | 0.3% | 0.5% | - |
| Basic premium per 0.1% humus increase | 200 €/ha | 220 €/ha | - |
| Additional premium/repayment per 0.1% humus increase/reduction at control investigation | 50 €/ha | 0 €/ha | - |
| Which alternative would you choose? | O | O | O |

**Table 10:** Choice Set 10

| Attributes | Humus Program A | Humus Program B | No Program Participation (opt-out) |
| --- | --- | --- | --- |
| Reference value for the measurement of the humus increase | Regional average of the last 3 years | Field-specific average of the last 3 years | - |
| Timing of the success investigation in years | 7 | 3 | - |
| Minimum increase in humus content at success investigation | 0.5% | 0.3% | - |
| Basic premium per 0.1% humus increase | 240 €/ha | 180 €/ha | - |
| Additional premium/repayment per 0.1% humus increase/reduction at control investigation | 0 €/ha | 50 €/ha | - |
| Which alternative would you choose? | O | O | O |

**Table 11:** Choice Set 11

| Attributes | Humus Program A | Humus Program B | No Program Participation (opt-out) |
| --- | --- | --- | --- |
| Reference value for the measurement of the humus increase | Field-specific average of the last 3 years | Field-specific humus content at the start of the program | - |
| Timing of the success investigation in years | 5 | 5 | - |
| Minimum increase in humus content at success investigation | 0.5% | 0.4% | - |
| Basic premium per 0.1% humus increase | 220 €/ha | 200 €/ha | - |
| Additional premium/repayment per 0.1% humus increase/reduction at control investigation | 50 €/ha | 0 €/ha | - |
| Which alternative would you choose? | O | O | O |

**Table 12:** Choice Set 12

| Attributes | Humus Program A | Humus Program B | No Program Participation (opt-out) |
| --- | --- | --- | --- |
| Reference value for the measurement of the humus increase | Field-specific humus content at the start of the program | Regional average of the last 3 years | - |
| Timing of the success investigation in years | 7 | 3 | - |
| Minimum increase in humus content at success investigation | 0.3% | 0.4% | - |
| Basic premium per 0.1% humus increase | 180 €/ha | 240 €/ha | - |
| Additional premium/repayment per 0.1% humus increase/reduction at control investigation | 0 €/ha | 50 €/ha | - |
| Which alternative would you choose? | O | O | O |
