# Supplementary material for: Farmers’ Willingness to Participate in a Carbon Sequestration Program – A Discrete Choice Experiment
Source: Environ Manage. 2024 Mar 21;74(2):332–49. doi: 10.1007/s00267-024-01963-9 (PMC11227454; doi:10.1007/s00267-024-01963-9)
Supplement: Supplementary file 5 — Online Resource 5 [file 267_2024_1963_MOESM5_ESM.docx]

**Online Resource 5**

# *Environmental Management*

# Farmers’ willingness to participate in a carbon sequestration program – a discrete choice experiment

Julia B. Block*, Michael Danne, Oliver Mußhoff

* Georg-August-University Göttingen

Department of Agricultural Economics and Rural Development

Platz der Göttinger Sieben 5

37073 Göttingen, Germany

[juliabarbara.block@uni-goettingen.de](mailto:juliabarbara.block@uni-goettingen.de)

Results of the multinomial logit model

**Table 1:** Estimation results of the multinomial logit model (N = 150)^a)^

|  | Coefficients | |
| --- | --- | --- |
| Variables | Model 1 | Model 2 |
| Program attributes |  |  |
| ASC | -1.104*** | -2.206*** |
| Field-specific average of the last 3 years^b)^ | 0.114** | 0.118** |
| Regional average of the last 3 years^b)^ | -0.235*** | -0.242*** |
| Timing of the success investigation in years | 0.104*** | 0.034 |
| Minimum increase in humus content at success investigation | -3.492*** | -2.839*** |
| Basic premium per 0.1% humus increase | 0.014*** | 0.015*** |
| Additional premium/repayment of 50€/ha per 0.1% humus increase/reduction at control investigation^c)^  Constant | -1.421***  -1.421*** | -0.272***  -1.421*** |
| Interaction terms |  |  |
| ASC x Motivation humus programs |  | 0.155** |
| ASC x Maximum subsidies |  | 0.144*** |
| ASC x Livestock density |  | 0.111*** |
| Timing of the success investigation x Farm size^d)^ |  | 0.042*** |
| Minimum increase x Farm size^d)^ |  | -0.537*** |
| Minimum increase x Risk attitude^e)^ |  | 0.030 |
| Goodness of fit |  |  |
| Participants/observations | 150/1,800 | 150/1,800 |
| Log-likelihood | -3,203.95 | -3,162.86 |
| AIC | 6,423.91 | 6,353.73 |

Single, double, and triple asterisks (*, **, ***) indicate statistical significance at 10%, 5% and 1% level.

^a)^ ASC = alternative specific constant; AIC = Akaike information criterion.

^b)^ Effect-coded variable; base level is ‘field-specific humus content at start of the program’.

^c)^ Effect-coded variable; base level is ‘0 €/ha’.

^d)^ Arable land in 100 ha.

^e)^ Risk attitude on a scale from 0 (strongly risk averse) to 10 (strongly risk seeking) according to Dohmen et al. (2011).
