# Supplementary material for: Farmers’ Willingness to Participate in a Carbon Sequestration Program – A Discrete Choice Experiment
Source: Environ Manage. 2024 Mar 21;74(2):332–49. doi: 10.1007/s00267-024-01963-9 (PMC11227454; doi:10.1007/s00267-024-01963-9)
Supplement: Supplementary file 6 — Online Resource 6 [file 267_2024_1963_MOESM6_ESM.docx]

**Online Resource 6**

# *Environmental Management*

# Farmers’ willingness to participate in a carbon sequestration program – a discrete choice experiment

Julia B. Block*, Michael Danne, Oliver Mußhoff

* Georg-August-University Göttingen

Department of Agricultural Economics and Rural Development

Platz der Göttinger Sieben 5

37073 Göttingen, Germany

[juliabarbara.block@uni-goettingen.de](mailto:juliabarbara.block@uni-goettingen.de)

Results from the Delta method for the confidence intervals of the WTA

**Table 1:** Farmers’ willingness to accept (Delta method; N=150)

|  | Model 1 | | Model 2 | |
| --- | --- | --- | --- | --- |
| Variables | WTA (€/ha) | Confidence intervals | WTA (€/ha) | Confidence intervals |
| Program attributes |  |  |  |  |
| Field-specific average of the last 3 years^a)^ | 9.93 | [1.57; 18.28] | 8.98 | [0.92; 17.03] |
| Regional average of the last 3 years^a)^ | -18.21 | [-30.71; -5.71] | -16.65 | [-28.79; -4.50] |
| Timing of the success investigation in years | 7.39 | [2.20; 12.59] | 0.50 | [-4.93; 5.93] |
| Minimum increase in humus content at success investigation | -323.76 | [-408.19; -239.33] | -439.70 | [-610.72; -268.68] |
| Additional premium/repayment of 50€/ha per 0.1% humus increase/reduction at control investigation^b)^ | -18.10 | [-26.15; -10.06] | -15.70 | [-23.36; -8.03] |
| Interaction terms |  |  |  |  |
| ASC x Motivation humus programs |  |  | 39.72 | [6.97; 72.46] |
| ASC x Maximum subsidies |  |  | 63.29 | [35.08; 91.50] |
| ASC x Livestock density |  |  | 46.53 | [17.76; 75.30] |
| Timing of the success investigation x Farm size^c)^ |  |  | 3.50 | [1.73; 5.27] |
| Minimum increase x Farm size^c)^ |  |  | -44.72 | [-71.04; -18.40] |
| Minimum increase x Risk attitude^d)^ |  |  | 31.62 | [8.27; 54.96] |

The WTA values were calculated for all statistically significant coefficients from Table 4 in the manuscript, which led to the exclusion of the ASC variable. (The price variable was set fixed.)

^a)^ Effect-coded variable; base level is ‘field-specific humus content at the start of the program’.

^b)^ Effect-coded variable; base level is ‘0 €/ha’.

^c)^ Arable land in 100 ha.

^d)^ Risk attitude on a scale from 0 (strongly risk averse) to 10 (strongly risk seeking) according to Dohmen et al. (2011).
